# Epigenetic State Changes Underlie Metabolic Switch in Mouse Post-Infarction Border Zone Cardiomyocytes

**DOI:** 10.3390/jcdd8110134

**Published:** 2021-10-22

**Authors:** Marie Günthel, Karel van Duijvenboden, Dennis E. M. de Bakker, Ingeborg B. Hooijkaas, Jeroen Bakkers, Phil Barnett, Vincent M. Christoffels

**Affiliations:** 1 Department of Medical Biology, Amsterdam Cardiovascular Sciences, University of Amsterdam, Amsterdam University Medical Centers, Meibergdreef 15, 1105 AZ Amsterdam, The Netherlands; m.gunthel@amsterdamumc.nl (M.G.); k.vanduijvenboden@amsterdamumc.nl (K.v.D.); i.b.hooijkaas@uva.nl (I.B.H.); p.barnett@amsterdamumc.nl (P.B.); 2 Hubrecht Institute-KNAW, University Medical Center Utrecht, 3584 CT Utrecht, The Netherlands; Dennis.deBakker@age.mpg.de (D.E.M.d.B.); j.bakkers@hubrecht.eu (J.B.); 3 Leibniz Institute on Aging-Fritz Lipmann Institute, 07745 Jena, Germany; 4 Department of Pediatric Cardiology, Division of Pediatrics, University Medical Center Utrecht, 3584 CX Utrecht, The Netherlands

**Keywords:** transcriptome, myocardial infarction, border zone, epigenetics, H3K27ac, nuclear RNA-sequencing

## Abstract

Myocardial infarction causes ventricular muscle loss and formation of scar tissue. The surviving myocardium in the border zone, located adjacent to the infarct, undergoes profound changes in function, structure and composition. How and to what extent these changes of border zone cardiomyocytes are regulated epigenetically is not fully understood. Here, we obtained transcriptomes of PCM-1-sorted mouse cardiomyocyte nuclei of healthy left ventricle and 7 days post myocardial infarction border zone tissue. We validated previously observed downregulation of genes involved in fatty acid metabolism, oxidative phosphorylation and mitochondrial function in border zone-derived cardiomyocytes, and observed a modest induction of genes involved in glycolysis, including *Slc2a1* (Glut1) and *Pfkp*. To gain insight into the underlying epigenetic regulatory mechanisms, we performed H3K27ac profiling of healthy and border zone cardiomyocyte nuclei. We confirmed the switch from Mef2- to AP-1 chromatin association in border zone cardiomyocytes, and observed, in addition, an enrichment of PPAR/RXR binding motifs in the sites with reduced H3K27ac signal. We detected downregulation and accompanying epigenetic state changes at several key PPAR target genes including *Ppargc1a* (PGC-1α), *Cpt2*, *Ech1*, *Fabpc3* and *Vldrl* in border zone cardiomyocytes. These data indicate that changes in epigenetic state and gene regulation underlie the maintained metabolic switch in border zone cardiomyocytes.

## 1. Introduction

Myocardial infarction (MI) remains one of the leading causes of mortality [1]. The acute cessation of oxygen and metabolite supply causes drastic hypoxia responses and metabolic changes. The oxygen shortage suppresses oxidative fatty acid (FA) metabolism and activates anaerobic glycolysis to reduce the consumption of the limited oxygen [2]. This acute phase is followed by death of irreplaceable cardiomyocytes and other cells in the ischemic region, immune responses and scar formation [3]. The infarct zone (IZ) is highly irregular in shape, consisting of necrotic tissue, infiltrating fibroblasts and immune cells, which will subsequently give rise to fibrous tissue. The remote myocardium (RM), distal from the infarct zone, is less affected by the ischemia. Cardiomyocytes of the border zone (BZ) surrounding the IZ are still viable, but severely affected by the ischemia, infiltrating immune cells and fibroblasts of the neighboring infarct zone [4]. BZ cardiomyocytes become hypertrophic and show decreased connectivity and interaction with neighboring cardiomyocytes and the extracellular matrix [5]. In the BZ, cardiomyocytes appear to undergo a dedifferentiative process, disassembling their sarcomeres, breaking down their sarcoplasmic reticulum and accumulating glycogen. Cardiomyocyte mitochondria also change in volume and shape, a phenotype that is often associated with chronic hibernation [4,6,7,8,9].

Recent transcriptome analyses revealed that following MI, gene expression profiles and phenotypes of the different cell types in the BZ region change and become much more heterogeneous [10,11,12,13,14,15,16,17,18]. The dedifferentiation of BZ cardiomyocytes and their reduction in metabolic activity following MI are seen to be accompanied by profound alterations in the transcriptome and in the epigenetic landscape [13]. When compared to cardiomyocytes from the RM, BZ cardiomyocytes display lower expression of genes involved in contractility, mitochondrial function, oxidative phosphorylation and fatty acid metabolism [13]. These transcriptomic and epigenetic alterations can be observed up to several weeks after MI, indicating the BZ cardiomyocytes are epigenetically maintained in a metabolically inactive and dedifferentiated state. The cardiomyocytes surrounding an injury in the zebrafish heart, which initiate proliferation to regenerate the heart, similarly dedifferentiate and downregulate genes for mitochondrial function and oxidative phosphorylation [19,20]. In addition, these cardiomyocytes increase glucose uptake and induce expression of glycolysis genes, and this metabolic switch was found to be essential for their proliferation during myocardial regeneration [19,21].

Previous studies using ATAC-seq revealed that the accessible chromatin landscape of BZ cardiomyocytes changed dynamically with thousands of chromatin regions gaining or losing accessibility [13]. ATAC-seq defines all classes of regulatory elements, irrespective of their function, including enhancers, promoters, silencers, insulators, boundary elements, etc. On the other hand, acetylation of K27 of histone H3 (H3K27ac) is predominantly enriched at sites flanking active promoters and enhancers [22]. The aim of this study was to further characterize the epigenetic landscape of BZ cardiomyocytes. We performed RNA-sequencing and cleavage under targets and release using nuclease (CUT&RUN)-sequencing [23] for H3K27ac on PCM-1 positive cardiomyocyte nuclei originating from the BZ and the LV. Most of the H3K27ac-marked putative enhancers that increased activity in BZ cardiomyocytes compared to controls were enriched for binding sites for AP-1 family of transcription factors, whereas the enhancers with reduced activity were enriched for binding sites for Mef2 transcription factors, validating previous ATAC-seq profiling data. However, enhancers with decreased H3K27ac association were also enriched for motifs recognized by the NR superfamily of transcription factors, including peroxisome proliferator-activated receptors (PPARs) and retinoid X receptor (RXR). RXR forms heterodimers with PPARα to regulate the expression of genes involved in fatty acid oxidation, inflammation and apoptosis [24,25,26]. We found that RXR/PPARα target genes such as *Cpt2* and *Ech1* are significantly downregulated in BZ cardiomyocytes. Together, our study indicates that changes in epigenetic state and gene regulation underlie the dedifferentiated and metabolically inactive state of border zone cardiomyocytes.

## 2. Materials and Methods

### 2.1. Myocardial Infarction and Tissue Isolation

Myocardial infarctions were performed as described previously [13]. Male mice of 8 weeks old from the FVB/N background (#001800, Janvier, France) were used to perform myocardial infarctions. Before surgery, mice received 0.075 mg/kg buprenofine (Covetrus, Cuijk, The Netherlands) subcutaneously. They were anesthetized with 4% isoflurane in 1 L/min oxygen and intubated during surgery. At the site of the chest opening, mice were shaved, and received local analgesia with lidocaine (2 mg/kg) and bupivacaine (3 mg/kg). Left thoracotomy was performed at the fourth intercostal space. An acute myocardial infarction was generated by permanent ligation of the left anterior descending coronary artery (LAD) by surpassing a BV130-5 6.5 mm taper point needle with a 8-0 Nylon wired (Ethicon, Johnson & Johnson, Amersfoort, The Netherlands) under the LAD, that was tied with a triple knot. The thoracotomy and skin were closed using a C-1 12 mm cutting needle with a 6-0 silicone coated brain silk wire (SOFSLIK, Medtronic, Eindhoven, The Netherlands). During surgery, anesthesia was maintained with the Minivent Mouse ventilator (Hugo Sachs Electronics, Harvard Apparatus) with 2% Isoflurane in 1 L/min oxygen. To maintain body temperature, mice were placed on a heating mat. Mice received analgesia up till 2 days by subcutaneous injection of Meloxicam (Covetrus, Cuijk, The Netherlands) at 0.2 mg/kg. The hearts were harvested 7 days after infarction. The left ventricle was isolated and dissected to obtain the border zone and remote myocardium. Mice were sacrificed by CO_2_ and cervical dislocation. 

### 2.2. Immunohistochemistry

For immunohistochemistry sections were deparaffinised in xylene, rehydrated through a graded series of ethanol and boiled for 5 min in unmasking solution (H3300, Vector) using a pressure cooker. Sections were stained with antibodies against PCM-1 (diluted 1:400, HPA023370, Atlas Antibodies, Bromma, Sweden, supplied by Bio Connect, Huissen, The Netherlands) and GLUT1 (diluted 1:100, GTX66489, Genetex). SYTOX Green was used (diluted 1:40,000, S7020, Invitrogen, supplied by Fisher Scientific, Landsmeer, The Netherlands) as a nuclear stain. The antibodies were visualized with the secondary antibodies Alexa647 (diluted 1:250, A-31571, Thermo Fisher) and Alexa555 (diluted 1:250, A-31572, Thermo Fisher). Sections were mounted in PBS:Glycerol (*v/v*). Pictures were taken with the Leica DM6000. Staining for p-PHD was performed using Anti-phospho-PDHE1-A type I (ser293) (ABS204, Merck, Supplier: Merck, Zwijndrecht, The Netherlands) as described before [19].

### 2.3. In Situ Hybridization

In situ hybridization was performed as described previously [27]. Briefly, sections were de-paraffinized and rehydrated to MilliQ. Sections were treated with Proteinase K (25530031, Invitrogen, Invitrogen, supplied by Fisher Scientific, Landsmeer, The Netherlands) and were pre-hybridized in hybridization buffer (50% formamide, 20X SSC, blocking reagent, 0.5 M EDTA, 10% CHAPS, heparine solution and 10 mg/mL yeast RNA) at 70 °C. Hybridization was performed overnight with dioxigenin (DIG) labeled probe against *Nppa* (forward: 5′gggcagagacagcaaacatc 3′, reverse: 5′ cacagtggcaatgtgaccaa 3′), Hk1 (forward: 5′ ggggatttcattgcactgga 3′:, reverse: 5′ tgtcgcagttcctccatgta 3′) and Pdk2 (forward: 5′ accggactctaagccagttc 3′, reverse: 5′ agatcttcttcaccgagggc). Probes were visualized using alkaline phosphatase-conjugated anti-DIG Fab fragments (11093274910, Roche, Supplier: Merck, Zwijndrecht, The Netherlands) and NBT/BCIP staining reagent (11681451001, Roche, Supplier: Merck, Zwijndrecht, The Netherlands). Sections were dehydrated and following mounted in Entellan (107961, Sigma Aldrich, Merck, Zwijndrecht, The Netherlands). Images were acquired with the Leica DM5000 microscope.

### 2.4. Isolation of Nuclei from Cardiomyocytes

Nuclei isolation was performed as described previously [13]. All steps were performed on ice. Snap frozen samples of the left ventricle, the micro-dissected border zone and remote myocardium were used. Samples were homogenized with the Ultra-Turrax homogenizer (IKA, Staufen, Germany) in 3 mL lysis buffer (10 mM Tris-HCL (pH8.0), 5 mM CaCl_2_, 2 mM EDTA, 0.5 mM EGTA, 1 mM DTT, 3 mM MgAc). 3 mL of lysis buffer containing 0.4% triton-X was added and the suspension was homogenized 10 times using a large and small pestle (Wheaton, Vancouver, Canada, supplier: Fisher Scientific, Landsmeer, The Netherlands). The solution was filtered through a 100 µm and 30 µm filter (Sysmex, CellTrics, Norderstedt, Germany). The filter were washed with 2 mL lysis buffer containing 0.2% triton-X. The solution was centrifuged for 5 min at 1000× *g* at 4 °C. The pellet was re-suspended in 500 µL staining buffer (2.5% BSA in PBS (pH 8.0 and 0.2% Igepal CA-630). The solution was incubated with rabbit anti-PCM-1 (siluted 1:1000, HPA023370, Atlas Antibodies) and Alexa 647 (diluted, 1:500, A-31573, Thermo Fisher) for 1 h. DAPI was added (0.001 mg/mL, Sigma-Aldrich, D9542, supplier: Merck, Zwijndrecht, The Netherlands). Sorting of PCM-1 positive nuclei was done on the BD Influx cell sorter (BD Bioscience, Vianen, The Netherlands). Nuclei were directly sorted into lysis buffer of the RNeasy plus micro kit (Quiagen) for subsequent RNA isolation or in 500 µL staining buffer for CUT&RUN-sequencing. 

### 2.5. RNA-Sequencing

RNA-sequencing from cardiac nuclei was performed as described previously [28]. 500 pg of RNA was used for cDNA preparation with the Ovation RNA-seq V2 kit (7102-08, Tecan, Leek, The Netherlands). Libraries were prepared with the UltraLow V2 kit (0344NB-08, Tecan, Leek, The Netherlands) and sequencing was performed on the HiSeq4000 system (Illumnia, San Diego, CA, USA) with 50 bp single-end reads. 

### 2.6. Differential Expression Analysis

Reads were mapped to the mm10 build of the mouse transcriptome using STAR [29]. Differential expression between groups was determined using the DESeq2 packaged based on using the negative binominal distribution [30]. *p*-values were corrected for multiple testing by using the false discovery rate of Benjamini- Hochberg (*p* < 0.05). Principal component analysis was performed with the DESeq2 package, using default parameters. Gene ontology (GO) term analysis was performed using DAVID v6.8 (https://david.ncifcrf.gov/ (accessed on 13 August 2021)). *p*-values indicated for the GO terms were corrected for multiple testing using Benjamini–Hochberg correction [31,32]. 

### 2.7. CUT&RUN-Sequencing

Cleavage under targets and release using nuclease (CUT&RUN) sequencing [23] was performed following the protocol from Hainer and Fazzio [33]. Approximately 50.000 nuclei were sorted by flow cytometry. Nuclei were collected in staining buffer and centrifuged for 5 min at 1000× *g* at 4 °C. The supernatant was discarded and the nuclei pellet was resuspended in 600 µL NE buffer (20 mM HEPES-KOH, pH 7.9, 10 mM KCl, 0.5 mM Spermidine, 0.1% Triton X-100, 20% glycerol, freshly added protease inhibitors), according to step 7 of the protocol. Nuclei bound to the Concanavalin A beads (86057-10, Polysciences) were incubated over night at 4 °C with anti- H3K27ac (diluted 1:200, 39133, Active Motif), The pA-MNase (1.75 μL) was diluted in 250 μL wash buffer and samples were incubated for 1 h at 4 °C.

For library preparation the NEBNext Ultra 2—DNA Library Prep Kit for Illumina was used (E7645S, E7335S, NEB), following the protocol according to an input of less than 50 ng DNA. For PCR enrichment of adaptor ligated DNA the protocol in [33] was followed (98 °C for 45 s, 14 times 98 °C for 15 s and 60 °C for 10 s, finished by 72 °C for 60 s). Elution of the library was performed in 20 µL 10 mM Tris-HCl. Samples were sequenced on the Illumnia HiSeq4000 paired-end sequencing 150 bp. 

### 2.8. Differential Acetylation Analysis

For differential acetylation analysis the reads were mapped to the mm10 build of the mouse genome using BWA [34]. The signal was distributed into bins of 500 bp using the BEDTools suite [35]. The background read level was determined per sample per 500 bp by taking the average read count in bins with less than 20 tags. The background level of tags was subtracted per bin per sample. To allow for differential peak calling between datasets, quantile normalization was applied using the deepTools2 suite [36]. Differential acetylation was assessed using the DESeq2 package based on a model using the negative binomial distribution [30]. *p*-values were corrected for multiple testing by using the false discovery rate of Benjamini-Hochberg (*p* < 0.05). Continuous bins with differential signal were subsequently merged together using the BEDTools suite. For promoter acetylation analysis the H3K27ac signal was distributed into promoter region bins defined as 2000 bp upstream and downstream of the canonical TSS (BioMart, vM3 annotation, mm10 build).

### 2.9. Overlap between H3K27ac and ATAC-Seq and Motif Analysis

H3K27ac data was intersected with ATAC-seq data obtained from 7 dpi FVB wildtype mice [13] using the BEDTools suite (v.2.30.0). To facilitate overlap analysis ATAC-peaks were increased 500 bp downstream and upstream. Motif enrichment analysis was performed on the 200 bp ATAC-seq summits using HOMER (v4.11) (http://homer.ucsd.edu/homer/), accessed on 20 May 2020 [37]. Motifs were classified as enriched when *p* < 1^−10^.

### 2.10. Overlap with ChIP-Seq

Raw ChIP-seq reads for Mef2 [38], c-Jun [39], PPARα [40] and RXR [41] were extracted from the Sequence Read Archives and mapped to the mouse genome (mm10) with BWA [34] as previously described [13].

## 3. Results

### 3.1. BZ Cardiomyocytes Downregulate Genes Involved in Lipid Metabolism

**Figure 1:**
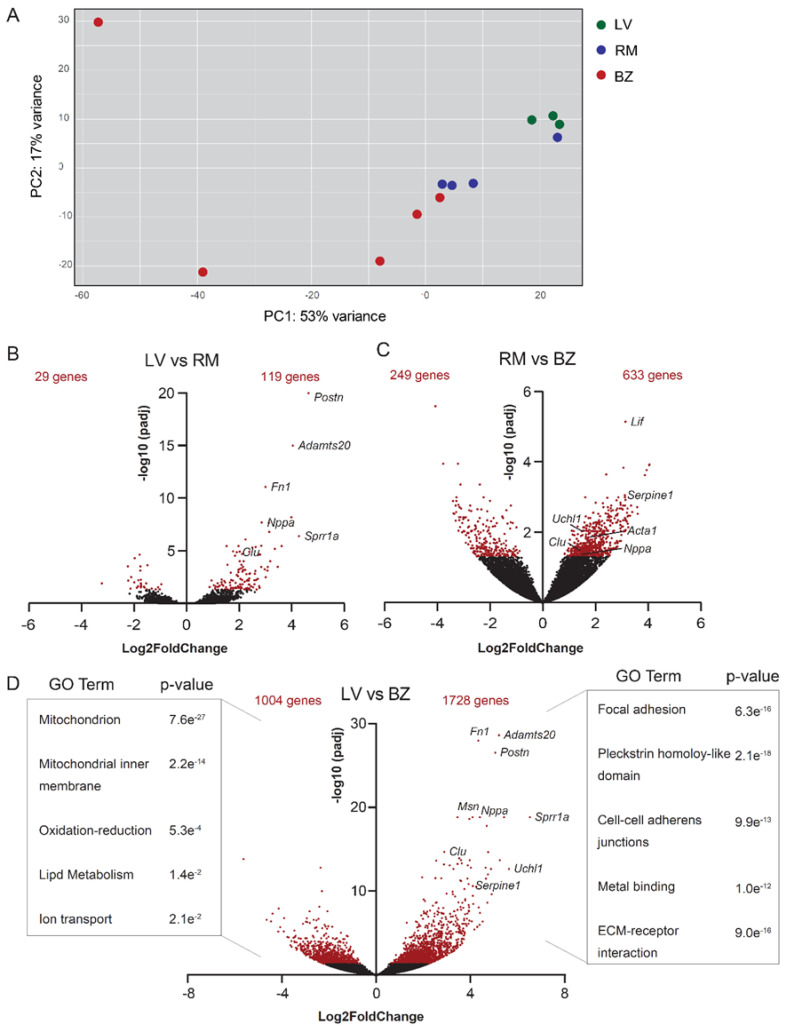
(**A**) Principal component analysis shows that samples from the left ventricle (LV) (*n* = 3) cluster together. Border zone (BZ) (*n* = 5) samples partially cluster with samples from the remote myocardium (RM) (*n* = 4) or do not cluster together. Green: LV, red: BZ, blue: RM. (**B**) Volcano plots showing nuclear transcripts of PCM1-positive cardiomyocytes that are differentially expressed between LV and RM and (**C**) RM and BZ. (**D**) Volcano plots showing nuclear transcripts of PCM1-positive cardiomyocytes that are differentially expressed between RM (*n* = 4) and BZ (*n* = 5). Red dots show significantly differentially expressed genes (*p* adjusted for multiple testing: false discovery date < 0.05). (**D**) Volcano plot shows significantly differentially expressed genes between BZ (*n* = 5) and LV (*n* = 3). Gene ontology analysis was performed on significantly higher and lower expressed genes of both groups.

We have previously compared the transcriptome of PCM-1-positive cardiomyocyte nuclei isolated from the BZ and RM [13]. To compare the transcriptomes of cardiomyocyte nuclei between cardiomyocytes from the BZ and the healthy left ventricle (LV), myocardial infarction was induced by ligation of the left descending coronary artery on 8-week-old male mice. At 7 days after the infarction, hearts were harvested and microdissected into BZ and RM. We compared the nuclear transcriptome of PCM1+ BZ cardiomyocytes (*n* = 5) with that of cardiomyocytes from the RM (*n* = 4) and the LV of age matched male mice (*n* = 3) (Table S1, Figure S1). Unsupervised principal component (PC) analysis revealed clustering of samples from the LV and one sample of the RM. Two samples from the BZ cluster together with samples obtained from RM. The remaining three BZ samples do not cluster together and show a high variance along the first and second PC (Figure 1A). Differences between the BZ samples may reflect variability in MI severity or response, and variation in the micro-dissected area, containing different ratios of BZ and RM cardiomyocytes. There are only around a hundred genes differentially expressed between the LV and the RM (Figure 1B). Of the several hundred genes differentially expressed between the BZ and the RM, the established BZ markers *Nppa*, *Acta1*, *Clu*, *Uchl1* and *Serpine1* are all represented as expected (Figure 1C) [13]. When compared to the cardiomyocyte nuclei of the control LV, those of the BZ differentially expressed 2732 genes (*p*-adj < 0.05; Figure 1D). Gene ontology (GO) analysis revealed that genes differentially expressed at higher levels are involved in processes of focal adhesion, cell-cell adherens junctions, as well as receptor interaction with the extracellular matrix. 

Genes that are lower expressed in the BZ CM nuclei are mainly involved in mitochondrial processes, lipid metabolism and ion transport. BZ cardiomyocytes also show significantly higher expression of genes known to be expressed by activated fibroblasts such as *Postn* and *Fn1* [12]. An important point to note here is that although sorting of PCM-1 positive nuclei from ventricular tissue highly enriches for cardiomyocyte nuclei [13,42], the very high number of fibroblasts and infiltrating immune cells in the BZ as compared to normal LV tissue may result in contamination of the PCM-1 positive cardiomyocyte nuclei population, causing inflated differences in gene expression between LV and BZ. 

### 3.2. Epigenetic State of Border-Zone Cardiomyocytes

**Figure 2:**
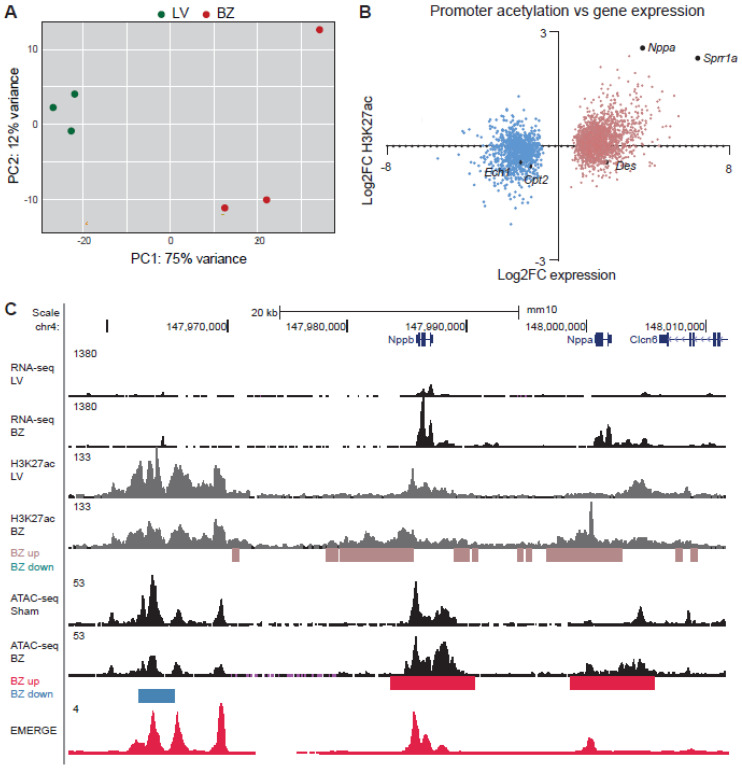
(**A**) Principal component analysis of samples from the left ventricle (LV) (*n* = 3) and the border zone (BZ) (*n* = 3) used for H3K27ac profiling by CUT&RUN-sequencing. (**B**) Scatterplot showing the relation between significantly differentially expression of genes between BZ and LV and differential acetylation of the transcription start site (*p* value adjusted for multiple testing: false discovery rate <0.05). (**C**) Representative UCSC browser view showing the *Nppa* and *Nppb* locus. ATAC-seq, assay for transposase-accessible chromatin using sequencing; CUT&RUN-seq, cleavage under target and release using nuclease-sequencing.

**Figure 3:**
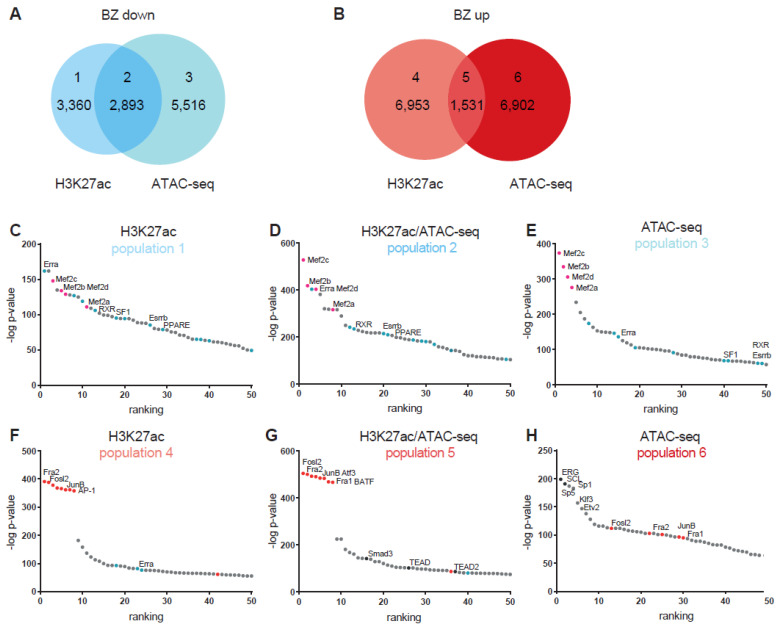
Venn diagram showing overlapping decrease (**A**) and increase (**B**) in H3K27ac and chromatin accessibility by ATAC-seq. Enriched motifs recognized by transcription factors for loci with decreased H3K27ac (**C**), decreased H3K27ac and chromatin accessibility (**D**), or decreased chromatin accessibility only (**E**)**.** Enriched motifs recognized by transcription factors for loci with increased H3K27ac (**F**), increased H3K27ac and chromatin accessibility (**G**), or increased chromatin accessibility only (**H**).

ATAC-sequencing at 7 dpi has shown that the chromatin accessibility of BZ cardiomyocytes changes with thousands of genomic regions gaining or loosing accessibility [13]. Regions gaining accessibility were significantly enriched for motifs recognized by AP-1 transcription factors (i.e. dimers of Jun, Fos, etc.). Less accessible sites were recognized by cardiomyocyte lineage-specific Mef2 transcription factors [13]. ATAC-seq identifies all regions of accessible chromatin, irrespective of the function of those sites. The accessible sites population therefore includes enhancers, promoters, silencers, insulators, boundary elements, etc. On the other hand, H3K27ac is predominantly enriched at sites flanking active promoters and enhancers [22]. We performed H3K27ac profiling of cardiomyocytes by CUT&RUN-sequencing in the BZ (*n* = 3) and the LV (*n* = 3) (Table S2). Principal component (PC) analysis shows clustering of the samples originating from the LV with the first component reflecting the origin of the tissue (Figure 2A). We analyzed H3K27ac at transcription start sites of 2663 differentially expressed genes and found that of 1736 genes that lost or gained H3K27ac signal in the BZ, that this directly correlated with the observed change in expression (Figure 2B and Table S2). The known BZ markers *Nppa* and *Nppb* displayed increased expression and increased H3K27ac at their promoters as expected (Figure 2C). We also cross-referenced the H3K27ac data with previously obtained data from ATAC-sequencing of LV and BZ cardiomyocytes [13]. When compared to the LV, 6253 regions showed less H3K27ac signal in BZ (Figure 3A). Of these, almost 3000 overlapped with sites of reduced accessibility. We detected increased BZ H3K27ac signal in 8484 regions. Of these, over 1500 overlapped with chromatin regions being also more accessible (Figure 3B). 

We used HOMER to identify enrichment of motifs of transcription factor binding sites at sites of differential accessibility or H3K27ac association (Table S3). Sites with decreased H3K27 acetylation were enriched for motifs recognized by Mef2 transcription factors, in accordance with the decreased accessibility of these motifs seen in the ATAC-seq (Figure 3C). In addition, we observed decreased acetylation of motifs recognized by the nuclear receptor (NR) superfamily of transcription factors, including estrogen related receptor alpha (Erra), retinoid X receptor (RXR) and elements for peroxisome proliferator-activated receptors (PPARs). These transcription factors are involved in the regulation of fatty acid metabolism and oxidative phosphorylation [25,26]. We obtained similar results for the population displaying a decrease in H3K27ac as well as diminished chromatin accessibility (Figure 3D). The population of less accessible chromatin in the ATAC-seq, but without a change in H3K27ac, displays mainly motifs recognized by the Mef2 transcription factors. Chromatin regions showing increased accessibility and increased H3K27ac association are enriched for binding sites of transcription factors that form AP-1 (Jun, Fos/Fra, Atf), showing that these putative regulatory elements are likely to be active in BZ cardiomyocytes (Figure 3E,F). The population that is only increased in accessibility as shown by ATAC-seq but does not show increased H3K27ac signal is mainly enriched for binding sites of members of the ETS (ERG, ETV), SCL, SP and KLF families of transcription factors, and to a lesser extend for binding sites of AP-1 transcription factors. 

### 3.3. Deregulation of PPAR Target Genes and Glycolysis Genes in BZ Cardiomyocytes

**Figure 4:**
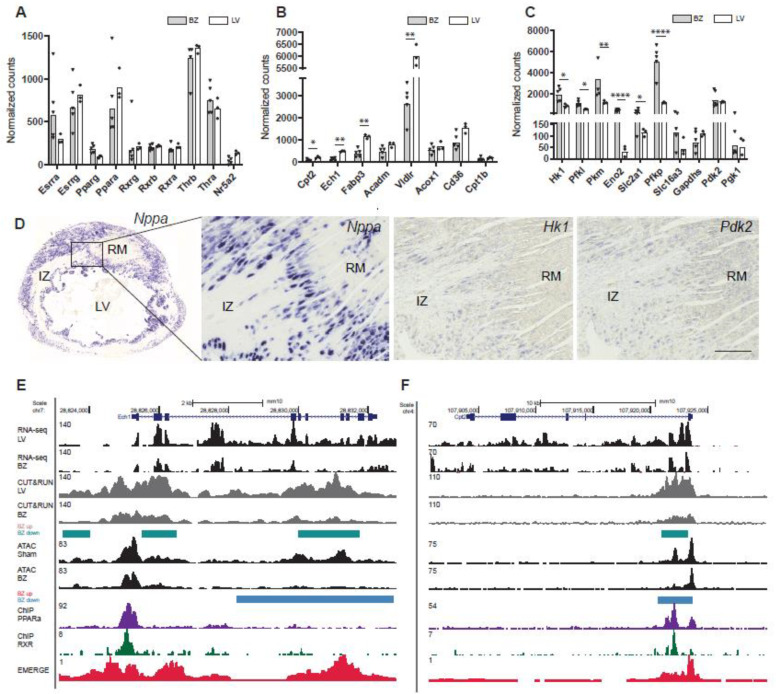
Average expression of NR transcription factors (**A**), PPAR target genes (**B**), and glycolytic genes (**C**) shown as normalized counts in border zone (BZ) samples (*n* = 5) and samples from the healthy left ventricle (*n* = 3). (**D**) Expression of *Hk1* and *Pdk1* is induced in *Nppa+* BZ cardiomyocytes, *Pdk1* expression is also visible in the RM as shown by in situ hybridization; scale bar 0.1 mm. UCSC genome browser views of (**E**) *Ech1* and (**F**) *Cpt2*. ATAC-seq, assay for transposase-accessible chromatin using sequencing; CUT&RUN-seq, cleavage under target and release using nuclease-sequencing; ChIP, chromatin immunoprecipitation sequencing; BZ, border zone; RM, remote myocardium; IZ, infarct zone; and LV, left ventricle. * *p* < 0.05, ** *p* < 0.01, **** *p* < 0.0001.

We next further explored the implications of the observation that NR binding sites are enriched in the population of chromatin sites with reduced H3K27ac signal. Expression levels of NR-transcription factors were not significantly changed between BZ and LV, although *Pparg* (encoding PPARγ) expression was slightly elevated in BZ cardiomyocytes (Figure 4A). We studied transcription levels of PPARα target genes that were reported to be downregulated in the failing heart and found that *Cpt2*, *Ech1*, *Fabpc3* and *Vldrl* were all significantly reduced in BZ cardiomyocytes [43] (Figure 4B). At the same time, BZ cardiomyocytes showed an increased expression of glycolysis genes *Hk1* (Hexokinase 1), *Pkm* (Pyruvate Kinase M1/2), *Pfkp* (Phosphofructokinase, Platelet) and *Eno2* (Enolase 2) and of *Slc2a1* (Solute Carrier Family 2 Member 1) important in the uptake of glucose into cardiomyocytes (Figure 4C). However, we did not observe these differences in expression when compared to the RM (Supplementary Figure S3A). Expression of *Hk1*, *Pdk2* and Slc2a1 (GLUT1) is induced in Nppa+ BZ cardiomyocytes, *Pdk2* expression is also visible in the RM (Figure 4D and Supplementary Figure S3B). Pyruvate dehydrogenase (PDH) converts pyruvate to Acetyl-CoA, thereby linking glycolysis to the TCA cycle (oxidative phosphorylation of pyruvate). Phosphorylation of PDH inactivates the enzyme complex, causing pyruvate to be diverted away from the TCA cycle, resulting in enhanced lactate production. We observed increased levels of phosphorylated PDH [19] in BZ cardiomyocytes compared to RM cardiomyocytes (Supplementary Figure S3C).

To investigate whether the changes in expression were associated with epigenetic state changes, we assessed the distribution of chromatin accessibility as well as H3K27ac signal and compared these with ChIP-seq data for PPARα [40] as well as RXR [41] (Figure 4E,F and Figure S1). The *Ech1* gene, encoding the enoyl CoA hydratase 1 (ECH1) is downregulated in BZ cardiomyocytes. The ECH1 enzyme plays a role in the mitochondrial β-oxidation pathway [44] and was previously identified as marker for the remote myocardium [13]. In BZ cardiomyocyte nuclei, H3K27ac association is decreased upstream of the gene locus as well as within the gene body, accompanied by a decrease in chromatin accessibility. ChIP-seq data for RXR and PPARα reveals binding sites for both transcription factors at the *Ech1* promotor (Figure 4E). The gene *Cpt2* encodes the carnitine palmytoiltransferase 2 (CPT2). CPT2 metabolizes acylcarnitines. Decreased levels of CPT2 were detected in ischemic hearts, leading to an increase of fatty acid intermediates and reactive oxygen species and thereby causing mitochondrial damage [45]. We observed a decreased chromatin accessibility overlapping a reduction in H3K27ac levels within the first intron of *Cpt2* (Figure 4F). These regions show binding sites for PPARα as well as RXR. The *Vldlr* gene encodes the very low-density lipoprotein receptor (VLDLR), that mediates the uptake of VLDL [46]. Chromatin accessibility is decreased for the upstream region and the first intron (Figure S4A). PPARα is associated with the differential H3K27ac regions including the promoter and first intron. 

**Figure 5:**
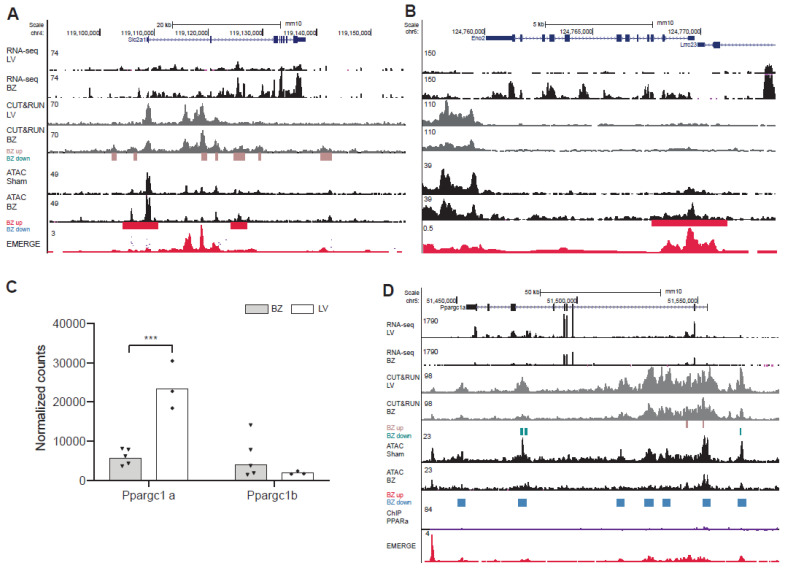
UCSC genome browser views of (**A**) *Slc2a1* and (**B**) *Eno2*. Average expression of *Ppargc1a* and *Ppargc1b* (**C**) shown as normalized counts in border-zone (BZ) samples (*n* = 5) and samples from the healthy left ventricle (*n* = 3). UCSC genome browser views of (**D**) *Ppargc1b*. ATAC-seq, assay for transposase-accessible chromatin using sequencing; CUT&RUN-seq, cleavage under target and release using nuclease-sequencing; ChIP, chromatin immunoprecipitation sequencing; BZ, border zone; and LV, left ventricle. *** *p* < 0.001.

Fatty acid binding protein 3 (FABP3) binds to long-chain fatty acids to facilitate lipid storage and metabolism, and overexpression of *Fabp3* caused apoptosis of cardiomyocytes after MI [47]. The whole locus of *Fabp3* becomes less accessible in BZ cardiomyocytes, however, H3K27ac signals are not decreased. There are several PPARα binding sites within the gene body and the upstream region flanking Fabp3, but no binding sites for RXR (Figure S4B). These data indicate that the downregulation of genes involved in fatty acid metabolism is driven by reduced enhancer-promoter activity in BZ cardiomyocytes, and that PPAR-RXR may be involved in this downregulation. In contrast, the induction of *Slc2a1*, *Eno2*, and *Pfkp* is associated with increased H3K27ac and accessibility of promoters and putative enhancers in these loci, suggesting that enhancer activity at these loci is increased (Figure 5A,B and Figure S4E). Signal for PPAR or RXR occupation was not detected in these loci. For *Hk1* and *Pkm* the landscape did not change consistently (Figure S4C,D).

In the heart, PPARs form a heterodimeric complex with RXR and recruit co-activators such as the peroxisome proliferator activated receptor alpha and beta (PGC-1α, PGC-1β) [48]. PGC-1α and -β control overlapping gene programs for cellular energy metabolism and are required for postnatal functional and metabolic maturation of the myocardium [48,49,50]. Target genes regulated by PGC-1 are involved in mitochondrial biogenesis, oxidative phosphorylation and fatty acid metabolism and decreased expression of PGC-1 is associated with a decrease in mitochondrial function [51]. We found that expression of *Ppargc1a* encoding PGC-1α is significantly reduced in BZ cardiomyocyte nuclei (Figure 5C). Expression of *Ppargc1b* is not significantly altered. In the BZ, the epigenetic state of both *Ppargc1a* and *Ppargc1b* is changed with several chromatin regions within the gene body being less accessible. In addition, several regions at both loci show decreased H3K27ac signal (Figure 5D and Figure S4F).

## 4. Discussion

When the coronary occlusion of an MI is not resolved, the myocardium downstream of the occlusion dies and an IZ is formed. The IZ is surrounded by a thin BZ, which contains surviving cardiomyocytes that are highly affected by the physiological condition of the heart and signals from the IZ. The BZ cardiomyocytes themselves are involved in post-MI arrhythmogenesis and play an active role in the healing process [13,52]. The MI BZ of the mammalian (human, mouse) heart shows similarities with the BZ of the injured fish heart [13,19,53]. Fish heart regeneration relies on proliferation of the pre-existing cardiomyocytes in the BZ [54]. Similarly, in mice with transgenically enhanced cardiac regenerative capacity, cardiomyocyte proliferation occurs mainly in the BZ cardiomyocytes [55,56,57,58]. Here, we focused on the transcriptional and epigenetic characterization of the cardiomyocytes in the BZ a week after permanent coronary artery ligation to gain further insight into the properties and potential of these cells. The BZ is comprised of several different cell-types, including endothelial cells, increased numbers of fibroblasts, infiltrating immune cells, and a small number of cardiomyocytes. All these cell types respond to the injury by inducing or repressing injury-responsive gene programs. This, along with the technical challenges of actually separating the different populations of cells, has hampered the characterization of specific cell types such as the cardiomyocytes in the BZ area. Thus far, most transcriptome data and epigenetic state data has been based on whole tissue preparations or cryosections of MI hearts, which has limited the detection of cardiomyocyte-specific responses [11,16,17,18,59]. The use of cell purification approaches or single cell sequencing can overcome these limitations, and have previously been deployed to gain insight into the transcriptomes of BZ cardiomyocytes [13] and cardiomyocytes of the left ventricle after ischemia-reperfusion [10,12]. Here, the purification of PCM-1+ cardiomyocyte nuclei from control left ventricular wall, RM and BZ tissue has allowed us to identify and characterize specifically their transcriptomes and, in addition, their epigenetic H3K27ac profiles. These data sets can be used as a resource and reference data set to explore and study novel aspects specifically relating to BZ cardiomyocytes. 

Our current data indicates that the transcriptome of cardiomyocytes of the BZ differs extensively from that of the control LV. The transcriptome of the RM cardiomyocytes was only modestly affected, indicating that the MI responses in RM and BZ cardiomyocytes is comparable but much more extensive in BZ cardiomyocytes. Almost 3 thousand genes were significantly differentially expressed between BZ and LV cardiomyocyte nuclei, and we focused on differential expression of genes involved in energy metabolism. During MI-induced ischemia, reduced oxygen delivery to the myocardium causes mitochondrial metabolic dysfunction and decreased ATP formation by oxidative phosphorylation. This stimulates an increase in glucose uptake and glycolysis, accompanied by lactate accumulation and decreased intracellular pH [60]. While the initial metabolic response to MI occurs at the level of protein activity, genes involved in mitochondrial activity and organization, energy metabolism (oxidative phosphorylation, fatty acid metabolism) were strongly downregulated in the BZ cardiomyocytes, and to a lesser extent in RM. This indicates that 7 and 14 days after MI, the reduction of aerobic energy production and fatty acid consumption have been firmly established in the gene programs in BZ cardiomyocytes [13]. Furthermore, we noted that expression levels of several genes involved in glucose uptake and metabolism are upregulated in BZ cardiomyocytes. This suggests that glucose metabolism preservation at least to some extent is supported by activation of the underlying gene program in BZ cardiomyocytes. In the fish heart, injury BZ cardiomyocytes switch energy metabolism from mitochondrial oxidative phosphorylation to glycolysis and lactate fermentation. This Nrg1/ErbB2 signaling induced switch is required for the proliferation of the BZ cardiomyocytes to regenerate the cardiac muscle [19]. Also in the mouse heart, activation of ErbB2 signaling caused an increase in cardiac expression of glycolysis genes and suppression of mitochondrial genes in normal and in MI hearts [19]. We also observed increased expression of the glycolysis gene *Pkm*, encoding pyruvate kinase muscle, in BZ cardiomyocytes, and to a lesser extent in RM cardiomyocytes. The M2 isoform of pyruvate kinase (PKM2) catalyzes a rate-limiting reaction in the glycolytic pathway but also modulates gene expression and contributes to tumorigenesis [61]. Intriguingly, a recent study demonstrated that cardiac *Pkm2* overexpression activates two separate and synergistic pathways (increased G6pd and interaction with β-catenin upregulating cell cycle genes) in cardiomyocytes to induce the cardiomyocyte cell cycle and cardiac regeneration after injury [62,63]. While the induced expression of native *Pkm* evidently is insufficient to induce proliferation of BZ cardiomyocytes, it may contribute to their predisposition to initiate proliferation after injury when additional cardiomyocyte cell cycle pathways are being activated, such as ERBB, YAP or hypoxia [63]. 

Previously, we found that the genome-wide chromatin accessibility patterns were changed in BZ cardiomyocytes compared to healthy cardiomyocytes. Accessibility reveals the degree to which nuclear protein complexes have physical access to the chromatinized DNA, irrespective of the function of the accessible site. H3K27ac marks the flanks of active enhancers and promoters [22], and therefore reveals the subpopulation of accessible sites that act as active enhancer or promoter. We assessed the genome-wide patterns of H3K27ac in BZ and control cardiomyocyte nuclei and found that thousands of putative enhancers and promoters gained or lost H3K27ac signal. The reduction in H3K27ac correlated with reduced expression of genes for oxidative phosphorylation and fatty acid metabolism, amongst others, indicating altered enhancer usage and epigenetic status underlies the changes in gene expression in BZ cardiomyocytes 7 days after MI. Accessible genomic sites associated with increased H3K27ac in BZ cardiomyocytes were highly enriched for motifs of binding sites for AP-1 transcription factors. This suggests that the AP-1 mediated transcription regulatory response leads to broad enhancer activation. Interestingly, sites that gained accessibility but not H3K27ac signal were only moderately enriched for AP-1 motifs, but highly enriched for binding sites of members of ERG/ETS, SP, ETV and KLF families of transcription factors. This suggests that these factors gain access to these sites in BZ cardiomyocytes, but that this does not lead to enhancer activation. In this respect, the recently identified function of KLF1 in rewiring of energy metabolism enabling cardiomyocyte proliferation and regeneration may be relevant in mammals [20]. Sites with reduced accessibility, reduced H3K27ac signal, or both, were enriched for MEF2 binding sites, suggesting that loss of MEF2 interaction with DNA occurs, and that this in many cases leads to reduced enhancer activity. Noteworthy, the sites of reduced H3K27ac signal are additionally enriched for motifs of binding sites for nuclear receptors (NRs) PPAR, RXR and ERRA. Previously, we did not detect these motifs as they are not obviously enriched in the population of sites with reduced accessibility [13]. 

In the heart, fatty acid metabolism is regulated by peroxisome proliferator-activated receptors (PPAR), members of the NR superfamily of transcription factors [26,64,65,66,67]. Conditions such as hypoxia were shown to lead to decreased PPARα expression and reduction of PPAR target gene expression causing cardiomyocytes to switch from fatty acid metabolism to glycolysis [67]. However, while we observed significant downregulation of PPARα target genes, we did not observe downregulation of PPAR family members or other NRs in BZ cardiomyocytes. Our data suggest that the reduction of expression of PPAR target genes is mediated by changed DNA binding activity of PPARs (and other involved NRs), leading to decommissioning of PPAR associated enhancers. The function of PPARs as regulators of transcriptional activity is complex and multi layered, and depends on their interaction with co-factors. PGC-1α is a co-factor of both PPARα-RXRα and of ERR (PPAR, RXR and ERR have similar binding motifs), and these complexes bind target gene enhancers and regulate energy metabolism [48,49,50,51]. *Ppargc1a*, encoding PGC-1α, is a target of PPAR-RXR and was strongly downregulated in BZ cardiomyocytes. We speculate that reduced concentrations of PGC-1α affects the formation of complexes with PPAR-RXR and ERR, respectively, influencing the association with particular enhancers. Furthermore, in the pathologically hypertrophic heart, PPARα forms a heterodimer with Sirt1 (Silent Information Regulator 1) instead of RXR, leading to altered complex function, altered enhancer usage and suppressed fatty acid metabolism [43,65]. However, while RXRα is downregulated and Sirt1 upregulated in the failing heart, supporting this switch, we did not observe any change in expression of the genes encoding RXR or Sirt family members in BZ cardiomyocytes. Interestingly, treatment of PPAR agonists or PPAR overexpression in the period around experimentally induced ischemia reperfusion or permanent MI shows varying outcomes, with some studies reporting reduced infarct sizes and improved cardiac function, whereas other studies report larger infarct sizes and impaired ventricular function [66]. PPARβ/δ activation has recently been proposed to be cardioprotective after ischemia reperfusion by reducing oxidative stress and maintaining mitochondrial function [68]. While the function of NRs like PPAR and ERR in the BZ cardiomyocytes is not well characterized, we hypothesize that PPAR agonist treatment may induce re-occupation of PPAR-dependent enhancers in BZ cardiomyocytes, which re-activates fatty acid energy metabolism in these cells, influencing their survival and function.

## Data Availability

The RNA-seq data and H3K27ac data has been deposited at the Gene Expression Omnibus under the following accession numbers: GSE183168 and GSE183095.

## Supplementary Materials

The following are available online at https://www.mdpi.com/article/10.3390/jcdd8110134/s1, Figure S1: Flow cytometry dot plot showing nuclei isolated from BZ tissue stained with DAPI and a secondary AB (Alexa647). Cardiomyocyte nuclei were identified by staining with anti-PCM-1 antibody bound by the secondary antibody Alexa-647. Figure S2: In situ hybridization at 7 dpi showing the expression of the cardiac marker Tnni3, the BZ marker Nppa, and Col1a1, marking the IZ. Scale bar, 1mm. Dpi, days post infarction; BZ, border zone, IZ, infarct zone; LV, left ventricle; RM, remote myocardium. Figure S3: (**A**) Average expression glycolytic genes shown as normalized counts in border-zone (BZ) samples (*n* = 5) and samples from the remote myocardium (RM) (*n* = 4). (**B**) Immunohistochemical staining of cTnI, GLUT1, and Sytox Green at 7 dpi. BZ, border zone; RM, remote myocardium; IZ, infarct zone. (**C**) Immunohistochemical staining of p-PDH at 7 dpi in RM and BZ. Scale bar, 10 μm. Dpi, days post infarction; RM, remote myocardium; BZ, border zone. Figure S4: UCSC genome browser views of (**A**) *Vldlr*, (**B**) *Fabp3*, (**C**) *Hk1,* (**D**) *Pfkm,* (**E**) *Pfkp,* and (**E**) *Ppargc1b*. Supplementary Tables S1–S3 (Excel files).
