# Supplementary figures and images for: Epigenetic State Changes Underlie Metabolic Switch in Mouse Post-Infarction Border Zone Cardiomyocytes
Source: J Cardiovasc Dev Dis. 2021 Oct 22;8(11):134. doi: 10.3390/jcdd8110134 (PMC8620718; doi:10.3390/jcdd8110134)

Supplemental Figure S1

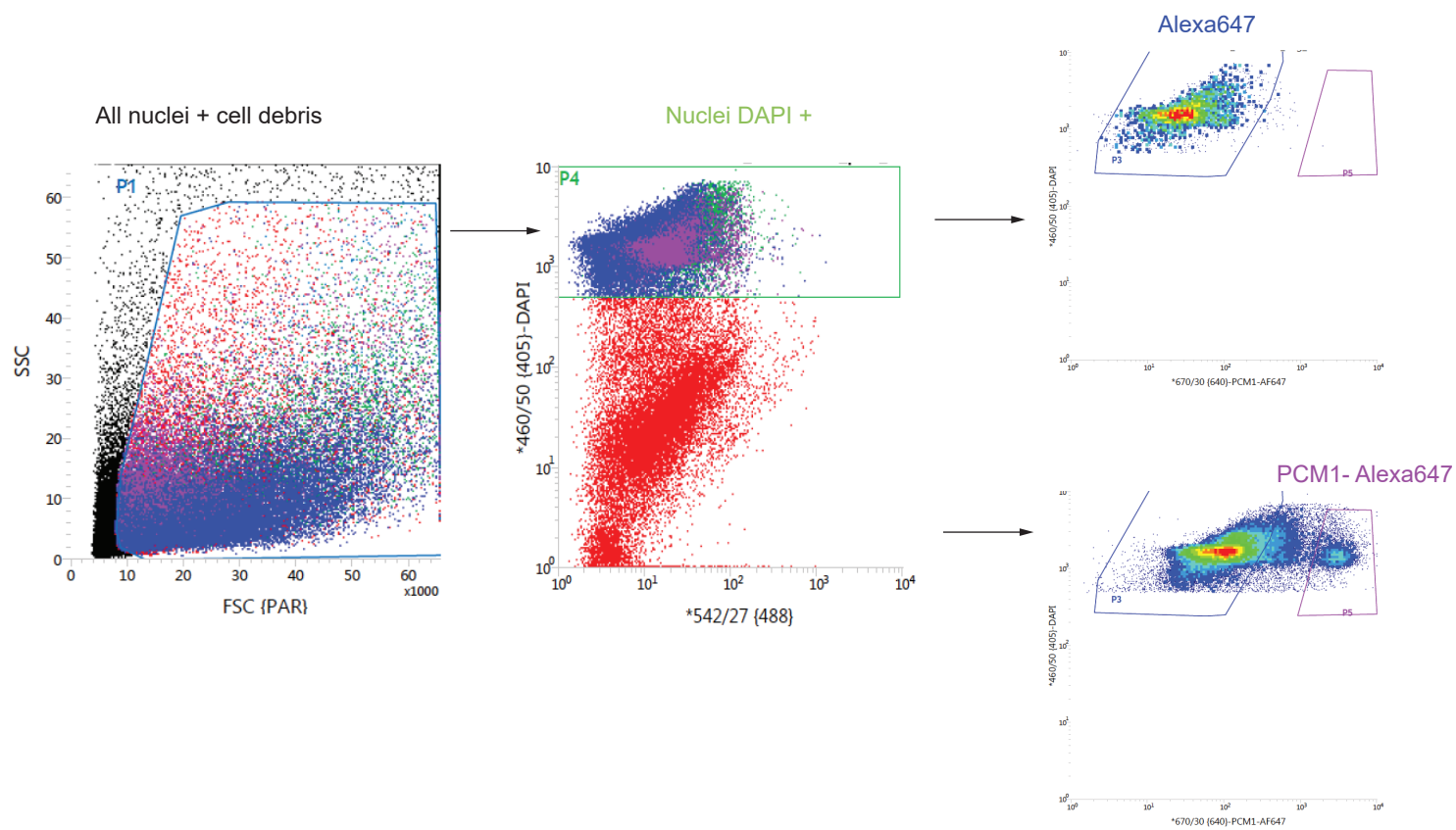

Supplement: Supplementary file 1 [file jcdd-08-00134-s001.zip › Suppl. Fig. 1.pdf]

Supplemental Figure S2

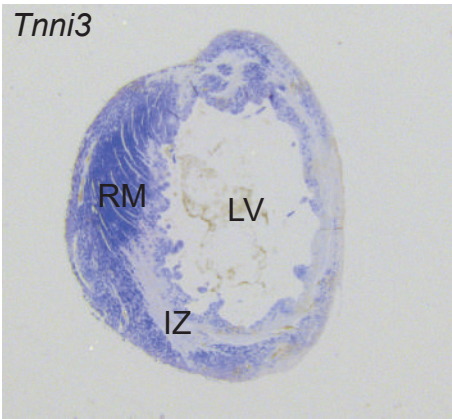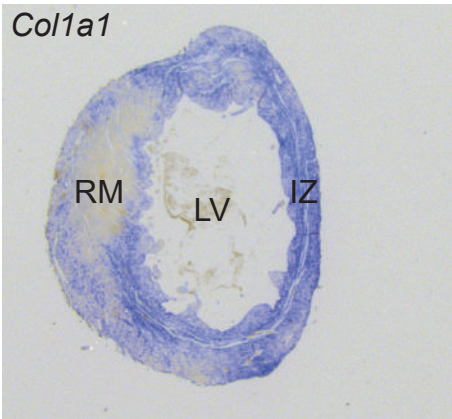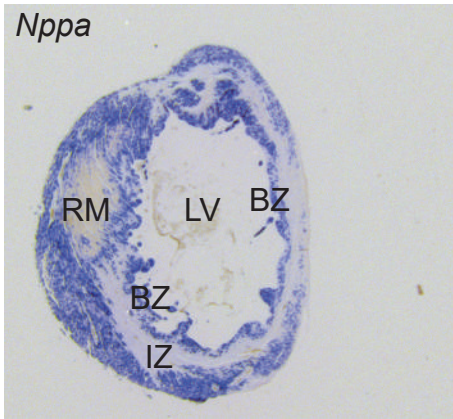

Supplement: Supplementary file 1 [file jcdd-08-00134-s001.zip › Suppl. Fig. 2.pdf]

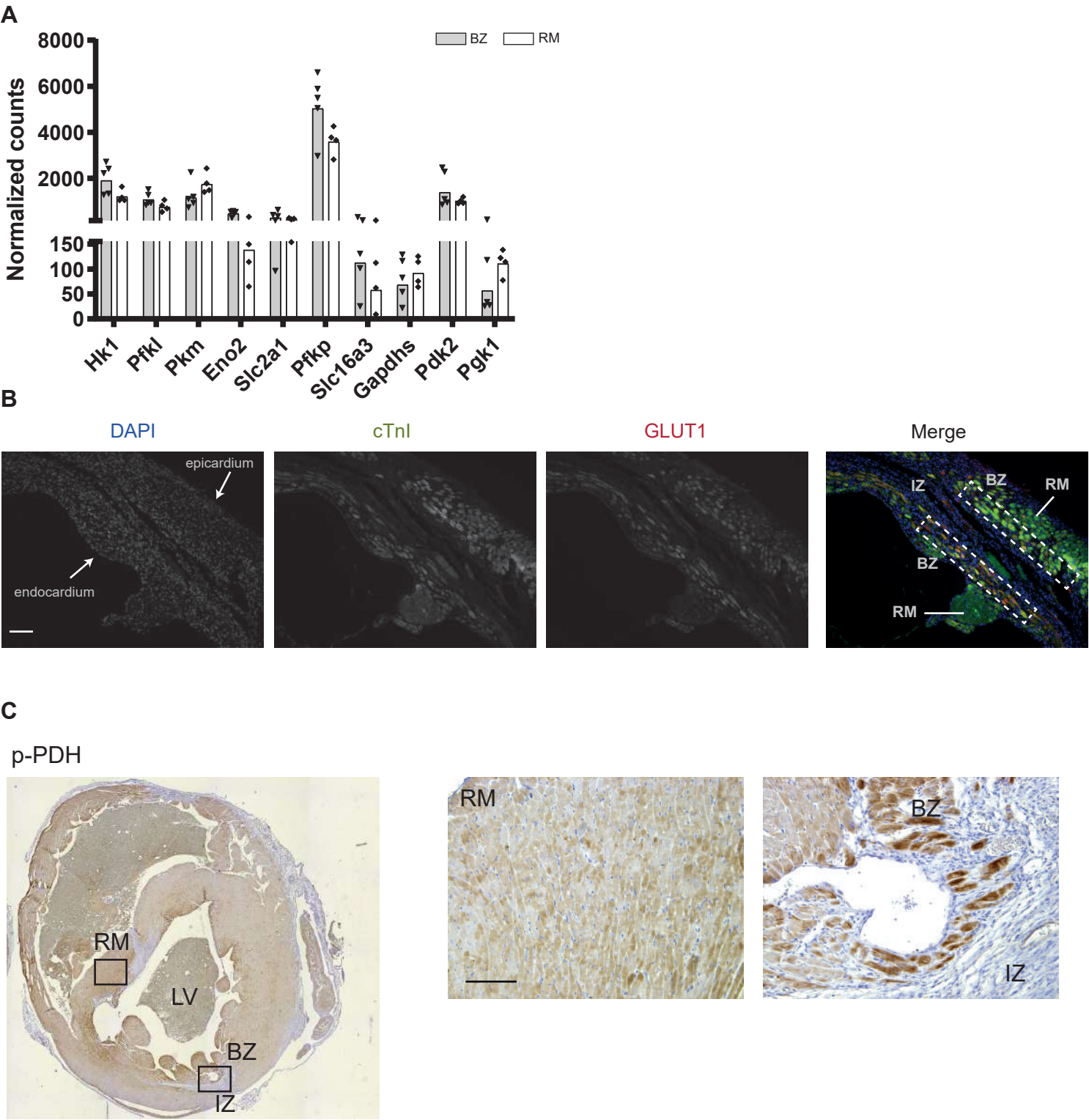

Supplement: Supplementary file 1 [file jcdd-08-00134-s001.zip › Suppl. Fig. 3.pdf]

Supplemental Figure S4

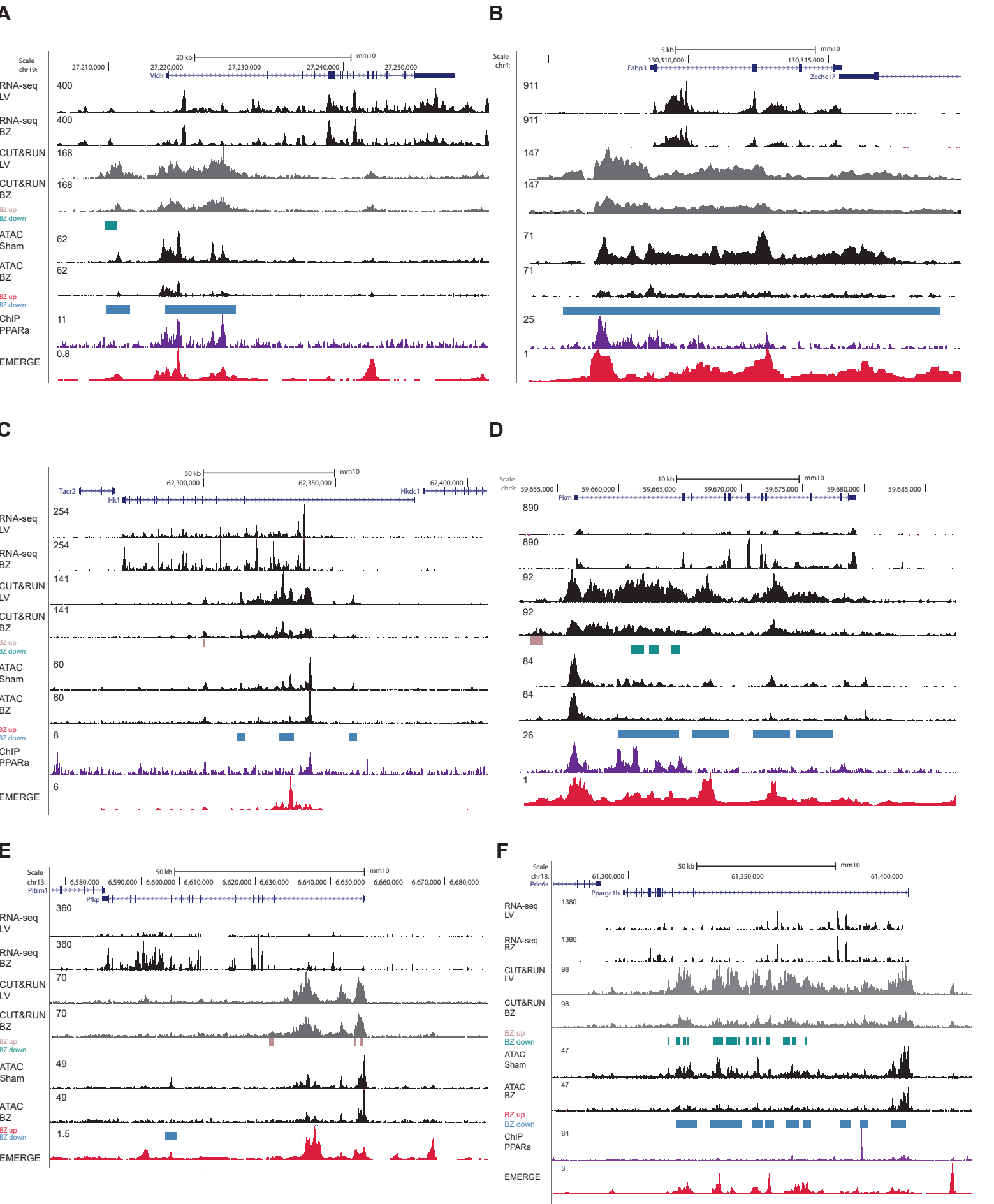

Supplement: Supplementary file 1 [file jcdd-08-00134-s001.zip › Suppl. Fig. 4.pdf]
